# Physicians' Perceptions of Intravenous Estrogen for the Management of Acute Abnormal Uterine Bleeding

**DOI:** 10.7759/cureus.99562

**Published:** 2025-12-18

**Authors:** Anne Tjaden Peiffer, Margaret Kistner, Abigail Otto, Elyse Schultz, Michael Wesolowski, Paula White, Linda C Yang

**Affiliations:** 1 Obstetrics and Gynecology, Loyola University Medical Center, Maywood, USA; 2 Biostatistics, Loyola University Chicago Stritch School of Medicine, Maywood, USA

**Keywords:** abnormal uterine bleeding, estrogen, physician preferences, survey, vaginal bleeding

## Abstract

Background

Acute abnormal uterine bleeding (AUB) is a common gynecologic complaint for which patients seek treatment. While many reasonable options exist for the management of AUB, the only Food and Drug Administration (FDA)-approved treatment option for acute AUB is intravenous (IV) conjugated estrogen. Our study aimed to investigate physicians' preferences for the management options of acute AUB and specifically examine their opinions regarding IV estrogen.

Methods

We created an electronic survey that was emailed to Obstetrics & Gynecology (OBGYN), Emergency Medicine (EM), and Internal Medicine (IM) physicians. Responses to the study were analyzed. Frequencies and percentages were reported to summarize survey responses. Cumulative or ordinal logistic regression models were used to estimate the effects of predictors on the odds of a higher likelihood or preference for prescribing IV estrogen for acute AUB.

Results

A total of 39 physicians responded to the survey. Only 6 (15.38%) indicated IV estrogen as their first-line choice for management of acute AUB. Only 13 participants (33.33%) indicated that they were comfortable prescribing IV estrogen. The most common indication for not prescribing IV estrogen was being unfamiliar with the typical regimen (18/26, 69.23%). Overall, OBGYN resident physicians were the most likely to feel comfortable prescribing IV estrogen.

Conclusions

Only a little over half of all participants correctly identified IV estrogen as being FDA-approved for the management of acute AUB. Approximately one-third of participants indicated that they were comfortable prescribing IV estrogen, while a near majority of participants indicated they were unfamiliar with the typical regimen. Future direction should include multidisciplinary educational initiatives regarding the management of patients with acute AUB.

## Introduction

Abnormal uterine bleeding (AUB) is a common reason that patients seek gynecologic care, both in the emergency department and outpatient setting. AUB encompasses bleeding between periods, heavier bleeding than normal, or irregular, unscheduled bleeding. It is estimated that approximately 20%-30% of people with a uterus will suffer from AUB during their life [1]. According to the American College of Obstetricians and Gynecologists (ACOG), acute AUB specifically “refers to an episode of heavy bleeding that, in the opinion of the clinician, is of sufficient quantity to require immediate intervention to prevent further blood loss” [2]. While chronic AUB may cause patients to seek care in an outpatient setting, acute AUB may cause patients to seek more urgent care in the emergency department. Patients may be treated in the emergency department and discharged home or, pending their clinical status, admitted to the hospital and managed by general medicine physicians or gynecologists. In fact, over 400,000 hospitalizations are thought to be due to AUB each year [3]. While the exact incidence of acute AUB is not well documented, it is a relatively frequent chief complaint at many institutions, including our tertiary care center. Abbas and Husain state that approximately 5% of all visits to the emergency department are related to AUB [4]. Acute AUB is not only bothersome for patients, but may ultimately pose life-threatening risks if it causes hemorrhage and acute blood loss anemia. It is also a major financial burden for patients, with one study finding that the "conservatively estimated annual direct and indirect economic costs of AUB were approximately $1 billion and $12 billion” [5]. Given the frequency and magnitude of this problem, gynecologists and other healthcare professionals who may care for patients with a uterus are tasked with the responsibility of managing AUB in the acute setting. 

Several management options exist for controlling acute AUB, which vary based upon patient presentation, comorbidities, ultrasound findings, and provider preference. In stable patients who do not require a surgical intervention, such as a dilation and curettage, medical management typically includes hormonal options that aim to stabilize the endometrium, although non-hormonal options also exist. Hormonal options include oral contraceptive pill (OCP) tapers, oral progestins, tranexamic acid (TXA), intramuscular medroxyprogesterone acetate (Depo Provera), and intravenous (IV) conjugated estrogen. With this breadth of options, the task of choosing which medication to initiate can feel daunting. Huguelet et al. examined pediatric providers (ED physicians, gynecologists, and adolescent medicine specialists) and demonstrated that even among this niche group, there was a wide variety in treatment plans [6]. Limitations in effective treatment may include providers’ lack of familiarity with specific regimens, access to certain formulations, or knowledge of appropriate pharmacologic agents. Matteson highlighted this in her study that surveyed OBGYNs and examined practice patterns in regard to AUB [3]. 

While multiple studies have demonstrated the efficacy of these various hormonal options in managing chronic AUB, currently, the only Food and Drug Administration (FDA)-approved management option for acute AUB is IV estrogen. While other medications may not be FDA-approved for the treatment of acute AUB, this does not mean they are not efficacious in this setting, and are used frequently, with good outcomes, in clinical practice. ACOG's Committee Opinion on acute AUB lists many hormonal options, such as OCPs and oral progestins, as reasonable treatment regimens [2]. However, IV estrogen has been studied specifically for use in acute AUB, originally in a randomized controlled trial by DeVore in 1982 [7]. This study examined 34 patients with AUB, with 18 randomized to receive IV estrogen. DeVore et al. found that after two injections of IV estrogen, 64% of patients in the treatment group had cessation of their bleeding, whereas only 11% of the control group did. 

At our institution, the management of acute AUB varies widely based upon provider preference. There are no standard algorithms or treatment protocols. Historically, IV estrogen has not been commonly used. During a three-year time period (2014-2016), IV estrogen was only ordered for seven patients. The lack of use may be due to the unfamiliarity with this agent, greater familiarity with other agents, or concerns regarding possible side effects of IV estrogen, including venous thromboembolism (VTE). Anecdotally, providers may be hesitant to use IV estrogen, given the concern for hypercoagulable effects in the high-risk population that typically presents to our tertiary care center. Our study aimed to examine physicians' preferences for the management of acute AUB and specifically examine their perception of IV estrogen used for this purpose. 

## Materials and methods

This survey study was conducted at Loyola University Medical Center in Maywood, Illinois. Our hospital is an academic medical center located in a busy metropolitan area with multiple residency programs. This study was submitted and reviewed by the Loyola University Medical Center Institutional Review Board (IRB). After approval was obtained (LU#213181), we created an electronic survey via RedCAP (Vanderbilt University, Nashville, TN, USA). We aimed to survey all physicians who may treat acute AUB in the hospital setting. Therefore, this survey was emailed to Obstetrics & Gynecology (OBGYN), Emergency Medicine (EM), and Internal Medicine (IM) physicians, as historically, physicians in these specialties manage and encounter AUB the most. Eligible participants included both resident and attending physicians employed at our institution. OBGYN attending physicians included any physicians who may treat AUB, such as general obstetricians and gynecologists, gynecologic oncologists, and minimally invasive gynecologic surgeons. Physicians were excluded if they were in a different specialty than was listed previously. 

The survey contained questions regarding participant training level (resident, attending), department (OBGYN, EM, or IM), and a short vignette describing a patient presenting to the emergency department with heavy vaginal bleeding. Questions regarding management preferences for AUB and perception of IV estrogen use were also included (Supplementary material 1). This survey was created for this study and has not yet been validated. Responses to the survey remained anonymous and were stored in the RedCAP database. Frequencies and percentages were reported to summarize survey responses in the overall sample, as well as among providers not comfortable prescribing IV estrogen for acute AUB. Cumulative or ordinal logistic regression models estimated the unadjusted or crude effects of department and training level on the odds of a higher likelihood or preference for prescribing IV estrogen for acute AUB. A subsequent multivariable model including both of these predictors estimated the adjusted effects of department and training level on this outcome. Wald 95% confidence intervals and chi-square p-values are reported for each odds ratio estimate. Proportional odds assumptions were evaluated using score tests and by comparing estimates from binary logit models for each dichotomization of the ordinal outcome. Statistical analyses were conducted using SAS Version 9.4 (SAS Inc., Cary, NC, USA).

## Results

A total of 39 participants completed the survey, with an estimated completion rate of ~15% to 19% (based on the estimated number of participants who received the survey). Twenty physicians identified as OBGYN (51.28%), 9 as EM (23.08%), and 9 as IM (23.08%), and 1 did not specify (2.56%). Twenty-two participants identified as residents (56.41%) and 17 as attending physicians (43.59%) (Table 1). Fifteen participants (38.46%) indicated OCP taper as their first-line management option for acute AUB, 15 (38.46%) indicated oral progestins, and 3 (7.69%) indicated TXA. Only six (15.38%) indicated IV estrogen as their first-line choice (Table 2). No participants indicated Depo Provera as their first-line management option. 

**Table 1:** Physician credentials, IV estrogen knowledge, and comfort ^†^1 respondent (2.56%) missing specialty. OBGYN: obstetrics and gynecology, FDA: Food and Drug Administration, AUB: abnormal uterine bleeding, IV: intravenous. Participants indicated their specialty, training level, and knowledge regarding whether a specific pharmacological agent was FDA-approved to treat acute AUB.  Frequencies and percentages are reported to describe categorical variable distributions.

Variable	n (%)
Specialty^†^	
Internal medicine	9 (23.08)
Emergency medicine	9 (23.08)
OBGYN	20 (51.28)
Training level	
Resident	22 (56.41)
Attending	17 (43.59)
Oral contraceptive pill taper FDA-approved for AUB	
Yes	26 (66.67)
No	13 (33.33)
Tranexamic acid FDA-approved for AUB	
Yes	14 (35.90)
No	25 (64.10)
Intravenous conjugated estrogen FDA-approved for AUB	
Yes	21 (53.85)
No	18 (46.15)
Intramuscular medroxyprogesterone acetate (Depot Provera) FDA-approved for AUB	
Yes	13 (33.33)
No	26 (66.67)
Oral progestin FDA-approved for AUB	
Yes	28 (71.79)
No	11 (28.21)
Comfortable prescribing IV estrogen for AUB	
Yes	13 (33.33)
No	26 (66.67)

**Table 2:** Pharmacological treatment preferences for acute abnormal uterine bleeding Survey respondents were asked to rank their pharmacologic treatment preferences for acute abnormal uterine bleeding on a scale from 1 to 5, with 1 being the most likely and 5 being the least likely. Frequencies and row percentages are reported to describe the distributions of clinician prescribing preferences for each pharmacological treatment option.

Pharmacological treatment, n (%)	1 (most likely)	2	3	4	5 (least likely)
Oral contraceptive	15 (38.46)	13 (33.33)	3 (7.69)	3 (7.69)	5 (12.82)
Tranexamic acid	3 (7.69)	3 (7.69)	15 (38.46)	9 (23.08)	9 (23.08)
IV estrogen	6 (15.38)	4 (10.26)	5 (12.82)	15 (38.46)	9 (23.08)
Intramuscular Depot Provera	0 (0.00)	5 (12.82)	12 (30.77)	6 (15.38)	16 (41.03)
Oral progestin	15 (38.46)	14 (35.90)	4 (10.26)	6 (15.38)	0 (0.00)

Despite IV estrogen being the only FDA-approved treatment modality for the management of acute AUB, only 21 participants (53.85%) identified it as such. Participants incorrectly identified the following therapies as being FDA-approved to treat acute AUB: OCPs (26/39, 66.67%), oral progestins (28/39, 71.79%), TXA (14/39, 35.90%), and Depo Provera (13/39, 33.33%) (Table 1). 

Only 13 participants (33.33%) selected that they were comfortable prescribing IV estrogen for acute AUB, indicating that two-thirds of participants were not comfortable doing so. The most common indication for not being comfortable with prescribing IV estrogen was being unfamiliar with the typical regimen (18/26, 69.23%). Two out of 26 participants (7.69%) stated they were not comfortable prescribing IV estrogen due to concern for its hypercoagulable effects. One participant (3.85%) indicated a lack of access to IV estrogen. Participants uncomfortable with prescribing IV estrogen did not cite its perceived ineffectiveness in stopping acute AUB as the reason (Table 3). 

**Table 3:** Reasons for discomfort with prescribing IV estrogen Survey respondents indicated reasons they were not comfortable prescribing IV estrogen. Frequencies and percentages are reported.

Reasons for prescribing discomfort	n (%)
Do not think IV estrogen is effective in stopping acute AUB	
Yes	0 (0.00)
No	26 (100.00)
Concerned with IV estrogen hypercoagulability risk	
Yes	2 (7.69)
No	24 (92.31)
Unfamiliar with IV estrogen and the typical regimen used	
Yes	18 (69.23)
No	8 (30.77)
No access to IV estrogen	
Yes	1 (3.85)
No	25 (96.15)
Prefer another management option based on experience/preference	
Yes	0 (0.00)
No	26 (100.00)

In cumulative logit regression models, the score test was significant for the unadjusted effect of department (p = 0.03) and for the multivariable model (p = 0.03), initially suggesting the proportional odds assumption may not have been valid for these models. However, the investigated binary logit models demonstrated that estimated effects did not vary substantially across each dichotomization of the ordinal outcome; therefore, the proportional odds assumption was considered satisfied. The score test was non-significant for the unadjusted effect of training level (p = 0.98), suggesting the proportional odds assumption was valid for the model. 

In examining physician specialty, there was no difference between IM and OBGYN physicians in regard to preference for prescribing IV estrogen to treat acute AUB (p = 0.32). EM physicians demonstrated significantly lower odds of a greater preference for prescribing IV estrogen to treat acute AUB compared to OBGYN physicians, and this effect remained statistically significant even after adjusting for training level (p = 0.01). The odds of a greater preference for prescribing IV estrogen to treat acute AUB were 90% reduced for EM physicians compared to OBGYN physicians after adjusting for training level (odds ratio (OR) 0.10; 95% confidence interval 0.02, 0.54). In examining training level alone, resident physicians demonstrated a greater preference for prescribing IV estrogen to treat acute AUB than attending physicians (p = 0.04). The odds of having a greater preference for prescribing IV estrogen to treat acute AUB for residents were 3.52 (95% CI: 1.05, 11.84) times those for attendings. This effect fluctuated very little but was no longer statistically significant after adjusting for department (OR: 3.52; 95% CI: 0.96, 12.83; p = 0.06). (Table 4). Overall, OBGYN resident physicians were the most likely to feel comfortable prescribing IV estrogen, whereas EM attending physicians were the least likely.

**Table 4:** Estimated effects of specialty and training level on IV estrogen prescribing preference for acute AUB ^*^Significant at α = 0.05 level. OR: odds ratio, CI: confidence interval. Participants’ specialty and training level were analyzed using simple and multivariable cumulative logit regression models to estimate the unadjusted and adjusted effects of predictors on the odds of a higher likelihood or preference for prescribing IV estrogen. Wald 95% CIs and Wald chi-square p-values are reported for each odds ratio estimate. The overall effect of the specialty predictor was significant (Type 3 p = 0.03), after adjusting for training level.

Predictor effect	Unadjusted OR (95% CI)	p-value	Adjusted OR (95% CI)	p-value
Specialty				
Internal medicine vs. OBGYN (Ref)	0.48 (0.11, 2.04)	0.32	0.60 (0.13, 2.70)	0.50
Emergency medicine vs. OBGYN (Ref)	0.11 (0.02, 0.55)	0.01*	0.10 (0.02, 0.54)	0.01*
Training level				
Resident vs. attending (Ref)	3.52 (1.05, 11.84)	0.04*	3.52 (0.96, 12.83)	0.06

## Discussion

While many studies have examined the management of chronic AUB in the outpatient setting, fewer have examined the management of acute AUB. Matteson et al. examined physicians' preferences for the management of non-emergent anovulatory AUB, non-emergent ovulatory AUB, and emergent AUB. In sampling over 800 OBGYN physicians who were members of ACOG, this study found that most physicians preferred an OCP taper as their first-line management of non-emergent AUB. In her vignette regarding emergent (acute) AUB in a current tobacco user, respondents preferentially selected dilation and curettage as their first-line management option. Of options for medical management, most participants selected oral progestins, while only 28% of respondents selected “estrogen single agent.” The survey also included questions regarding the efficacy of certain treatment options for AUB and found that “only 25% (n = 86) answered at least two out of three questions correctly” [3]. In a study by Lee et al., 100 physicians in South Korea were sampled in regard to the management of AUB (primarily chronic). Most respondents also indicated OCPs as their first-line treatment preference [8]. To our knowledge, our study is the first to examine multiple specialties (other than OBGYN) and their preferences for management of acute AUB. Similar to Matteson et al. and Lee et al., our study demonstrated that the most preferred initial management option for acute AUB (across all specialties) was an OCP taper. 

The preference for OCPs may be due to their commonality and familiarity across a wide range of prescribing specialties, as well as their long-standing safety and efficacy data. When we examined the comfort of physicians prescribing IV estrogen to treat acute AUB, we found there was no difference between IM and OBGYN. However, OBGYN physicians felt more comfortable with IV estrogen than EM physicians. Perhaps unsurprisingly, OBGYN residents felt more comfortable than other groups, likely from their exposure and education surrounding IV estrogen use during their training. 

There is limited data examining practitioners’ attitudes on IV estrogen. IV estrogen is rarely used at our institution, and we hypothesized that this may be due to providers' concern about the associated risks of VTE. However, among the providers who did not feel comfortable prescribing IV estrogen (~66% of respondents), only 7.6% identified hypercoagulable effects as their reason. Approximately 70% of those who did not feel comfortable stated that they were unfamiliar with the typical regimen. This indicates that perhaps more providers would feel comfortable ordering IV estrogen if a specific protocol or electronic medical record order set were established for ease and safety of prescribing. In fact, Close et al. demonstrated that incorporating an AUB treatment algorithm improved knowledge for pediatric residents treating AUB in an emergency room setting [9]. This indicates that an algorithmic approach to such a complex problem, such as AUB, may provide a more streamlined approach to treatment and result in better patient care. 

Most physicians in our study were unable to identify that IV estrogen is the only FDA-approved treatment for acute AUB. While this may be reasonable (physicians may not be aware of all drugs that are or are not FDA-approved), given the fact that IV estrogen is effective and safe at controlling acute AUB, it is important that physicians have some knowledge and feel comfortable prescribing this medication. Any physician who treats AUB should have a basic understanding of all medications that may be used for this purpose, along with knowledge regarding dosing and side effect profiles. Understanding which medications are FDA-approved and which are used off-label is also important so as to better counsel patients and understand which medications to prescribe and when. 

There are several strengths to our study. One strength includes the incorporation of multiple specialties in our analysis, including OBGYN, internal medicine, and emergency medicine physicians. Our study is also unique in its focus on the management of acute AUB, rather than the more commonly studied chronic AUB. In addition, our study not only examined physicians' preferences for management options, but also attempted to assess physicians’ rationale for their prescribing preferences in order to identify gaps in knowledge or other addressable barriers. There are several limitations to our study. First, we only examined providers at our own institution rather than at multiple sites. Because we included physicians from multiple specialties, there is the possibility of bias in treatment preferences based upon specialty focus and frequency of treating AUB (e.g., OBGYN physicians will encounter and manage AUB more frequently than IM physicians). Our study did not include all pharmacological options for management of acute AUB, such as non-steroidal anti-inflammatories (NSAIDs), oral estrogens, or surgical management such as dilation and curettage, uterine tamponade, or hysterectomy. Like many studies that include surveys, our response rate was lower than desired. Because we emailed the survey link to the department coordinators, who then forwarded the email to both residents and attendings, it is difficult to assess who actually received the link, and therefore, the exact response rate is difficult to determine, but is estimated to be around 15%. As a result of the low response rate, our study did not meet its target sample size and had a final sample size that was lower than desired. A consequence of the low sample size is that the study may not have had adequate statistical power to detect the regression model estimates that we generated. However, we feel that these estimates we generated are still valuable and can, for instance, be used to power future, larger-scale prospective studies. 

Future directions may include editing our survey to include more background and descriptive information of participants, as well as adding more questions regarding other treatment options for acute AUB, as listed above. The survey could also be distributed to providers at different institutions, ideally nationwide, to examine the differences in practice patterns depending upon location. Furthermore, at our institution, a multidisciplinary educational session including OBGYN, EM, and IM providers who care for patients with AUB should be implemented to teach how and when to prescribe IV estrogen. While IV estrogen is not the only way to treat acute AUB, it has been proven to be effective, and therefore should be recognized as a valid management option for patients presenting with this complaint. While it is difficult to standardize treatment for AUB, given its complexity in presentation and its various options for treatment, an established protocol or algorithm may provide a more streamlined approach to treatment and overall improved patient care. 

## Conclusions

Our study aimed to examine physicians' preferences for the management of acute AUB, as well as physicians' perception of IV estrogen. Nearly half of the respondents failed to correctly identify IV estrogen as the only FDA-approved treatment modality for acute AUB. Furthermore, the majority of respondents did not feel comfortable prescribing IV estrogen for acute AUB. While there are many efficacious treatment options for acute AUB, it is important for physicians to be aware of, and feel comfortable prescribing, all approved medications in order to appropriately treat patients. Given the infrequent use of IV estrogen at our institution, as well as providers’ limited comfort with prescribing it, there exists a need for further education surrounding its administration, dosage, and side effects. Our study has identified an area for improvement that will in time hopefully result in improved patient care.

## Appendices

Supplementary material 1 

**Table 5:** Survey questionnaire

Survey	
1. Please select the following which best describes your specialty:	A. OBGYN
	B. Emergency medicine
	C. Internal medicine
2. Please select the following which best describes you:	A. Resident
	B. Attending
3. Ms. Smith is a 32 y/o G1P1001 with no past medical history presenting with vaginal bleeding. She reports that she has been bleeding for 2 days. The bleeding started out fairly light but has gotten heavier. She is passing clots. She is currently using 3 pads/hour. She feels slightly dizzy but has no other symptoms. Her LMP was 18 days ago. Her periods are normally regular and her cycle is ~28 days. She is hemodynamically stable, her hemoglobin is 7.5. Her last sexual activity was 3 months ago and her pregnancy test is negative. Pelvic ultrasound displays no polyps or fibroids. You diagnose acute abnormal uterine bleeding. Please rank the following pharmacological options in the order in which you would most likely prescribe them to help control this patient’s bleeding (1 = most likely, 5 = least likely)	A. Oral contraceptive pill taper
	B. Tranexamic acid
	C. Intravenous conjugated estrogen
	D. Intramuscular medroxyprogesterone acetate (Depot Provera)
	E. Oral progestin (i.e., medroxyprogesterone acetate, norethindrone)
4. According to your knowledge, which of the following are FDA-approved pharmacologic management options for acute abnormal uterine bleeding? (Please mark all that apply)	A. Oral contraceptive pill taper
	B. Tranexamic acid
	C. Intravenous conjugated estrogen
	D. Intramuscular medroxyprogesterone acetate (Depot Provera)
	E. Oral progestin (i.e., medroxyprogesterone acetate, norethindrone)
5. Do you feel comfortable prescribing IV estrogen to a patient with acute abnormal uterine bleeding?	A. Yes
	B. No
6. If you marked “no,” please mark all of the following that apply regarding your answer.	A. I do not think IV estrogen is effective in stopping acute abnormal uterine bleeding
	B. I am concerned with the hypercoagulability risk associated with IV estrogen
	C. I am unfamiliar with IV estrogen and the typical regimen used
	D. I do not have access to IV estrogen
	E. I prefer another management option based on experience/preference
7. If you marked yes, please select the following answer that best describes you.	A. I am comfortable treating a patient with IV estrogen
	B. I am comfortable treating a patient with IV estrogen but would prefer a gynecology consultation before prescribing this medication

## References

[1] Dyne PL, Miller TA (2019). The patient with non-pregnancy-associated vaginal bleeding. Emerg Med Clin North Am.

[2] Committee on Gynecologic Practice (2013). Management of acute abnormal uterine bleeding in nonpregnant reproductive-aged women. Obstet Gynecol.

[3] Matteson KA, Anderson BL, Pinto SB, Lopes V, Schulkin J, Clark MA (2011). Practice patterns and attitudes about treating abnormal uterine bleeding: a national survey of obstetricians and gynecologists. Am J Obstet Gynecol.

[4] Abbas T, Husain A (2021). Emergency department management of abnormal uterine bleeding in the nonpregnant patient. Emerg Med Pract.

[5] Liu Z, Doan QV, Blumenthal P, Dubois RW (2007). A systematic review evaluating health-related quality of life, work impairment, and health-care costs and utilization in abnormal uterine bleeding. Value Health.

[6] Huguelet PS, Buyers EM, Lange-Liss JH, Scott SM (2016). Treatment of acute abnormal uterine bleeding in adolescents: what are providers doing in various specialties?. J Pediatr Adolesc Gynecol.

[7] DeVore GR, Owens O, Kase N (1982). Use of intravenous Premarin in the treatment of dysfunctional uterine bleeding--a double-blind randomized control study. Obstet Gynecol.

[8] Lee JY, Lee DY, Song JY, Lee ES, Jeong K, Choi D (2015). A national survey of gynecologists on current practice patterns for management of abnormal uterine bleeding in South Korea. Int J Gynaecol Obstet.

[9] Close AG, Sequeira GM, Montano GT (2019). Improving the evaluation and management of abnormal uterine bleeding in female adolescents presenting for emergency care. J Pediatr Adolesc Gynecol.

